# European region of the WCPT statement on physiotherapy in primary care

**DOI:** 10.1017/S1463423619000811

**Published:** 2019-11-04

**Authors:** Jill Long

**Affiliations:** European Region WCPT Rue de Pascale, 36 Brussels B-1040, Belgium

**Keywords:** Physiotherapy, Primary Care

## Abstract

This statement has been produced by the European Region of the World Confederation for Physiotherapy (ER-WCPT) to promote the role of the physiotherapy profession within primary care, to describe the health and economic benefits to health systems and populations of having a skilled, appropriately resourced and utilised physiotherapy workforce in primary care services, and to illustrate how different models of physiotherapy service delivery are contributing to these health and cost benefits.

## Physiotherapy in primary care

Primary health care is a comprehensive, interdisciplinary, patient-centred and community-oriented approach to health care. A primary care service is the patient’s first point of entry into the health care system and the continuing focal point for all their health care needs (Aafp.org., 2019).

Physiotherapists’ qualifications and their ability to screen (Boissonnault and Ross, 2012), diagnose and provide appropriate treatment or referral (Ludvigsson and Enthoven, 2012) for musculoskeletal, neurological cardiorespiratory, paediatric and other conditions have positioned them as important providers of quality health care within the primary care team. Physiotherapists’ participation in primary care contributes to addressing the growing demand for health services and the increasing costs associated with delivering these services across Europe. Physiotherapy is an effective, comparatively low-cost high-value option to meet this rising demand (Mitchell and De Lissovoy, 1997; Ojha *et al*., 2014; Marks *et al*., 2016a). The safety and efficacy of physiotherapy in primary care is well established (Swinkels *et al*., 2014; Mintken *et al*., 2015) as is its effect on reducing high and over-utilisation of other medical services (Bornhöft *et al*., 2015).

## Health and economic benefits on physiotherapy in primary care

Models of physiotherapy service delivery vary throughout European countries, ranging from prescribed treatments by a medical doctor to direct access or self-referral, where patients access physiotherapy services directly, without the need to see a doctor first. Self-referral is the norm in countries like the Netherlands, Norway, Sweden and the UK, and self-referral to physiotherapy in primary care has been fully evaluated and is recommended by the National Institute for Health and Care Excellence (NICE) in the UK. It has been shown to reduce waiting times, put patients in control, enabling them to manage their condition and live more independently, and improve health outcomes by preventing acute problems from becoming chronic and reducing long-term pain and disability (The Scottish Government, 2015; The Chartered Society of Physiotherapy, 2019), reduce time off work (Department of Health, 2008), reduce costs for the NHS in the UK and reduce rates of medication prescribing (Holdsworth *et al*., 2007).

### Physiotherapy services have been implemented and studied in various settings such as general practice, orthopaedic outpatient clinics, emergency departments and primary care

In general practice, musculoskeletal conditions account for around 1 in 5 of all GP appointments (CSP, 2017). The majority of these patients do not need to see a GP and can be effectively managed by a physiotherapist, freeing millions of GP appointments for other patients each year (Ludvigsson and Enthoven, 2012). Support for direct access to physiotherapy is strongly expressed by both service users and clinicians (Holdsworth and Webster, 2004). Fewer patients require multiple GP visits for their musculoskeletal disorder, referral to specialists/external examinations, sick leave recommendations or prescriptions during the following year compared to a GP-assessed group (Bornhöft *et al*., 2015).

The effectiveness of physiotherapists’ management of musculoskeletal conditions within orthopaedic outpatient services has been well studied; the diagnoses and triage recommendations for patients with hip and knee disorders made by physiotherapists are similar to the orthopaedic surgeons while patient satisfaction is significantly higher for physiotherapist care (Desmeules *et al*., 2013). Patients’ perception of quality of care in a physiotherapist-led musculoskeletal service is significantly higher than that in a standard practice group. This model of care seems to meet patients’ expectations and result in a greater intention to follow advice and instructions for self-management (Samsson *et al*., 2016). A physiotherapist with additional prescribing and injection training makes decisions analogues to those of an orthopaedic surgeon at initial consultation for orthopaedic shoulder pain, including the safe identification of patients for subacromial injection (Marks *et al*., 2016b). This model of physiotherapist-led service delivery is safe, effective and can be delivered in primary care settings.

The effectiveness of physiotherapists’ caseload management in emergency departments (EDs) has been well studied; Patients attending EDs with certain conditions can be effectively managed by physiotherapists and referred to appropriate primary care services without the need for admission to an acute hospital. This can contribute to the management of certain conditions within primary care rather than in acute hospital. When comparing similar diagnostic groups, physiotherapists are significantly more time-efficient than ED physicians while managing a great deal of their caseload independently and safely (de Gruchy *et al*., 2015). Patients with musculoskeletal problems in a primary practitioner physiotherapy service in Australia had improved waiting times and length of stay relative to Australian College of Emergency Medicine and Department of Health targets (Gill and Stella, 2013). A multidisciplinary primary care team project, including physiotherapy, for patients with long-term conditions in the UK has been shown to reduce attendances at EDs by 15%, reduce emergency admissions to hospital by 55%, improve patients’ function by 75% and reduce their anxiety by 50%, and reduce costs of care as compared to treatment in the acute hospital setting (Edwards, 2014). The findings of these studies show that primary care physiotherapy provides an opportunity to shape patient-centred care, improve access and offer quality care on the most appropriate level, with associated health and economic benefits.

### There is evidence to support the management of certain conditions within primary care physiotherapy services rather than in acute hospitals, and some examples are described

The Health Information and Quality Authority in Ireland reviewed the evidence for performing shoulder arthroscopy for a number of shoulder conditions (Health Information and Quality Authority, 2014a), knee arthroscopy for knee osteoarthritis, arthroplasty for knee osteoarthritis and arthroplasty for hip osteoarthritis (Health Information and Quality Authority, 2014b) and recommended a trial of physiotherapy in the primary care setting for at least three months before referring for a surgical opinion as the more clinical and cost-effective management.

NICE guidelines on low back pain management (National Institute for Health and Care Excellence, 2016) recommend reassurance, advice to keep active, guidance on self-management, exercise programmes and manual therapy – these are the core skills of a primary care physiotherapist which position them as significant members of the primary care workforce dealing with low back pain. Direct access to experienced specialist physiotherapists is effective in managing spinal conditions conservatively and identifying surgical candidates appropriately (Wood *et al*., 2016).

There is growing evidence that targeted exercise prescription can improve the health of people in the community living with cancer and has a cost benefit (Storic *et al*., 2013; Broderick *et al*., 2014; Titz *et al*., 2016) in terms of recurrence, progression and survival (Holmes *et al*., 2005; Holick *et al*., 2008; Ibrahim and Al-Homaidh, 2011; Ballard-Barbash *et al*., 2012; Chlebowski, 2013; Arem *et al*., 2014). Prescribed exercise can also contribute towards better survival rates by addressing post-diagnosis weight gain. Weight gain has been linked to poorer survival rates in breast cancer (Arem *et al*., 2015).

Falls in older people are one of the main reasons older people are admitted to EDs. Physiotherapy to restore and maintain functional mobility, bone health, strength and balance in older people can significantly reduce their risk of falls and their risk of injury if they fall. In 2014, the Chartered Society of Physiotherapy Falls Prevention Economic Model found that if everyone over the age of 65 years at risk of falling was referred to physiotherapy within primary care, this could reduce the number of patients who currently end up in emergency hospital department following a fall by 225 300, saving the UK health service £331 million every year. They calculated that every £1 spent on physiotherapy produces a £1.50 return on investment (The Chartered Society of Physiotherapy, 2014).

Falls and Chronic obstructive airways disease (COPD) among older people are two of the main reasons older people are admitted to hospital and require social care in the community. They are also two areas where primary care physiotherapy can have a major impact. Physiotherapy-run pulmonary rehabilitation classes for COPD patients in the UK resulted in these patients being less likely to be admitted to hospital. Those admitted spent less time in hospital and were 26% less likely to be readmitted following discharge (Seymour *et al*., 2010).

A paediatric physiotherapist and an orthopaedic surgeon had substantial to almost perfect agreement for diagnosis, treatment and follow-up in children referred for gait abnormalities (Miller *et al*., 2016), showing that physiotherapists can contribute to the effective management of such conditions in primary care.

In November 2017, the UK Government announced plans to extend fit note certification beyond GPs to a wider group of health care professionals, including physiotherapists, psychiatrists and senior nurses, to better identify health conditions and treatments to help workers go back into their jobs faster, and to assist people with disabilities to enter or remain at work. Fit notes are designed to help patients develop a return to work plan tailored to their individual needs (GOV.UK, 2017).

These are examples of the conditions that can be effectively managed by physiotherapists in the primary care setting. There are many other conditions and populations that physiotherapists can manage effectively within primary care, such as neurological conditions and women’s health issues, but these are less well described in the literature.

## What is physiotherapy?

Physiotherapists are autonomous health professionals who are responsible for developing, maintaining and restoring human performance throughout the lifespan using evidence-based practice. They treat or prevent conditions associated with pain, injury, disease or other impairments. Physiotherapists empower patients and their carers to manage their condition outside clinical settings and to retain their independence.

Physiotherapy education in most European countries enables physiotherapists to practice autonomously in their assessment, diagnosis, management and discharge of patients. Trained to identify serious pathology, many physiotherapists are qualified to undertake medical screening of patients and to refer accordingly. In some countries, trained physiotherapists are authorised to administer injections or prescribe specific drugs.

## What is the ER-WCPT?

The ER-WCPT is a non-profit, non-governmental organisation that represents the physiotherapy profession at European level. The organisation has a membership of 39 physiotherapy associations, 1 from each of the European countries, including all the EU member states, EEA countries and all the EU applicant countries, representing over 180 000 physiotherapists in Europe. It is one of the five regions that make up the WCPT, a global organisation representing physiotherapists and physical therapists worldwide. The WCPT claims exclusivity to the professional names ‘physical therapy’ and ‘physiotherapy’ and abbreviations of same, as the sole preserve of persons who hold qualifications approved by its member organisations. The titles are used interchangeably throughout the world and refer to the same profession. In Europe, ‘physiotherapy’ is the title most commonly used and is therefore used in this paper to encompass both titles.

ER-WCPT is a member of the European Forum for Primary Care and supports its aim of improving the health of the population by promoting strong primary care.
